# PPAR-α Modulators as Current and Potential Cancer Treatments

**DOI:** 10.3389/fonc.2021.599995

**Published:** 2021-03-23

**Authors:** Yan Tan, Mina Wang, Ke Yang, Tiange Chi, Zehuan Liao, Peng Wei

**Affiliations:** ^1^School of Traditional Chinese Medicine and School of Life Sciences, Beijing University of Chinese Medicine, Beijing, China; ^2^Beijing Key Laboratory of Acupuncture Neuromodulation, Department of Acupuncture and Moxibustion, Beijing Hospital of Traditional Chinese Medicine, Capital Medical University, Beijing, China; ^3^The First Clinical Medical School, Beijing University of Chinese Medicine, Beijing, China; ^4^School of Biological Sciences, Nanyang Technological University, Singapore, Singapore; ^5^Department of Microbiology, Tumor and Cell Biology (MTC), Karolinska Institutet, Biomedicum, Stockholm, Sweden

**Keywords:** PPAR-α, PPAR-α modulators, cancer, cancer treatment, PPAR-α agonists, PPAR-α antagonists

## Abstract

Cancer is one of the leading causes of mortality worldwide. PPAR modulators may hold great potential for the management of cancer patients. Indeed, PPARs are critical sensors and regulators of lipid, and they are able to promote eNOS activation, regulate immunity and inflammation response, and affect proliferation and differentiation of cancer cells. Cancer, a name given to a group of diseases, is characterized by multiple distinctive biological behaviors, including angiogenesis, abnormal cell proliferation, aerobic glycolysis, inflammation, etc. In the last decade, emerging evidence has shown that PPAR-α, a nuclear hormone receptor, can modulate carcinogenesis via exerting effects on one or several characteristic pathological behaviors of cancer. Therefore, the multi-functional PPAR modulators have substantial promise in various types of cancer therapies. This review aims to consolidate the functions of PPAR-α, as well as discuss the current and potential applications of PPAR-α agonists and antagonists in tackling cancer.

## Introduction

Cancer is one of the worldwide health problems, and the well-established risk factors involving genetic susceptibility, ionizing radiation, infections, smoking, insobriety, unhealthy diet, sedentary lifestyle, obesity, and other carcinogenic environmental exposures, promote the prevalence of cancer (1–5). It is believed that more than 20 million new cancer cases annually are projected to occur by 2025. Lung cancer remains to be the leading cause of morbidity and mortality globally (6). Besides, the American Cancer Society has demonstrated that cancer is the second leading cause of death in the United States, with expected 1,762,450 new cancer cases and 606,880 cancer deaths in 2019 (7). In fact, the morbidity and mortality of multiple kinds of cancer declined over the past decade due to decreases in known risk factors, effective screening, early detection, and better treatments in high-income countries. In contrast, cancer rates increased in low- and meddle- income countries, resulting from increases in smoking, unhealthy diet, lack of physical activity and infections (8). For example, death rates in the poorest countries were 2-fold higher in terms of cervical cancer, and 40% higher for men with lung and liver cancers from 2012 to 2016, compared with the most developed countries (7). Moreover, since cancers are characterized by mutations, and cells in our body inevitably mutate as we age, some of the mutations trigger formation of malignancy (9, 10). Considering the characteristics of cancer, two major therapeutic schemes have been applied clinically. One is genotype-directed precision oncology, targeting specific genomic abnormalities of various types of cancer to provide individual treatment. Another is antitumor immunity, that is, therapies targeting at the components of tumor microenvironment, especially the immune system (11–16).

Peroxisome proliferator-activated receptors (PPARs) are members of three ligand-inducible transcription factors, which belong to the nuclear receptor super-family. PPARs play an important role in regulating the expression of a variety of genes regarding the metabolic homeostasis of glucose and lipid, adipogenesis, and inflammation (17, 18). In mammals, there are three subtypes of PPARs: PPAR-α, PPAR-γ, and PPAR-β/δ, possessing varying expression levels in different tissues, biological effects, and ligand affinities (19). PPAR-α is mainly expressed in brown adipose, skeletal muscle, heart, liver, and intestinal mucosa tissues, adjusting glucose and lipid metabolism and homeostasis, inflammation, immune response, and angiogenesis (20, 21). Therefore, due to the vital metabolic modulating roles and excellent druggability of PPARs, PPAR agonists and antagonists have been employed for the therapy of a number of diseases, such as dyslipidemia, type 2 diabetes, cardiovascular diseases, obesity, cancer, and other metabolic diseases (22). PPAR agonists promote the transcription of target genes, leading to structural adjustment in the heterodimerized PPAR (with retinoid X receptor). However, PPAR antagonists do not change receptor conformation, compete or not compete with other ligands (23).

Mounting evidence has been accumulating in effects of PPAR-α and PPAR-γ in carcinogenesis, which show overlapping functions in metabolism and inflammation modulation. Yet, respective distinctions in specificity and potency of PPAR-β/δ has been conflicting. Apart from the regulation of cancer cell proliferation and differentiation regulated by PPAR modulators (both agonists and antagonists), which have been widely investigated, their pleiotropic roles in cancer encompasses realms of metabolism and inflammation are highly associated with cancer types and specific microenvironment as well (24). To provide a more focused insight, this review aims to only discuss recent findings on the biological functions, as well as current and potential applications of the agonists and antagonists of PPAR-α in cancer treatments.

## Functions of PPAR-α

**Figure 1:**
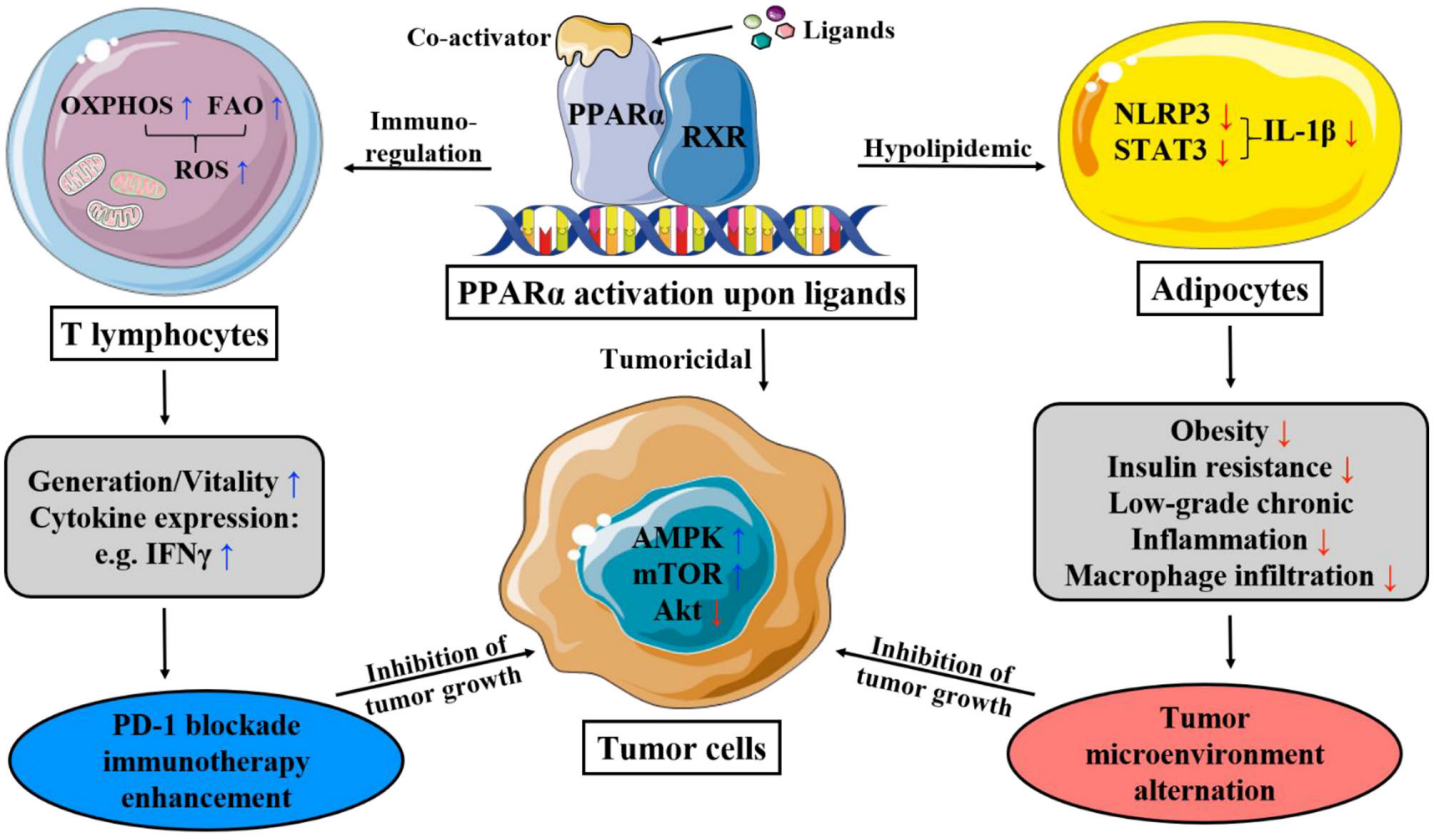
This figure illustrates the main mechanism of PPAR-α that lipid and immunity regulation lead to apoptosis of cancer cells. OXPHOS, oxidative phosphorylation; FAO, fatty acid oxidation; ROS, reactive oxygen species; PD-1, programmed death-1; AMPK, AMP-activated protein kinase; mTOR, mammalian target of rapamycin; STAT-3, signal transducer and activator of transcription-3; NLRP3, nucleotide-binding domain, leucine-rich-containing family, pyrin domain-containing-3.

PPAR-α is universally expressed in an organism, whose RNA expression level lacks tissue specificity and that of protein is particularly significant in tissues that require fatty acid oxidation as a source of energy, primarily metabolic tissues like brown adipose tissue, liver, heart, and kidney (25). The anti- and pro-tumorigenesis properties of PPAR-α are intricately intertwined in the field of the metabolism of lipid, glucose, and amino acid, as well as inflammation, cell proliferation, and apoptosis (Table 1 and Figure 1).

**Table 1:** Summary of functions and mechanisms of PPAR-α.

**Functions**	**Mechanisms**
Lipid regulation (22, 26–30)	1. Promotion of β-oxidation 2. Serum triglyceride clearance and HDL increase 3. Induction of hepatic lipogenesis
Glucose regulation (31, 32)	1. Alleviation of glucose disposal (in skeletal muscle) 2. Amelioration of insulin resistance 3. Inhibition of aerobic glycolysis (in cancer cells)
Amino acid regulation (30)	1. Decrease of amino acid metabolism rate as a part of starvation response
Inflammation regulation (22, 31, 33)	1. Downregulation of gene expression of inflammatory cytokines, e.g., IκB, IL-6, TNFα 2. Downregulation of the AP-1 and NF-κB pathways 3. Interaction with glucocorticoid receptor α or estrogen receptor
Cardiovascular benefit (34)	1. Activation of endothelial nitric oxide synthase (eNOS)
Anti-tumorigenesis effect (22, 29, 32, 35–48)	1. Inhibition of angiogenesis 2. Prioritization of FAO to glycolysis and disruption of the balance of glucose and lipid metabolism to inhibit ATP production 3. Accumulation of ROS and mitochondrial damage 4. Induction of apoptosis and ferroptosis 5. Control of DNA methylation 6. Preservation of endocannabinoids 7. Inhibition of melanogenesis 8. Support on the generation and vitality of T cells, the conversion to T-eff cells, and the prevention of the apoptosis of cytotoxic T lymphocytes (CTLs)
Pro-tumorigenesis effect (49–60)	1. Decreased antioxidant capacity and CPT1A pattern expression^*^ 2. Promotion of CYP1B1, which is involved in the bioactivation of procarcinogens 3. Induction of liver cancer in rodents^**^ 4. Promotion of self-renewal ability and sphere-formation rate of cancer stem cells (CSCs) 5. Suppression on the proliferation and vitality, and the induction of apoptosis of T cells^*^

* *Contradictory evidence*.

** *Evidence absent in humans; ROS, reactive oxygen species; FAO, fatty acid oxidation; AP-1, activator protein-1*.

### Regulation of Lipid Metabolism

PPAR-α maintains lipid metabolism and homeostasis via the modulation of genes of lipoprotein lipase, apolipoprotein (e.g. APOA1, APOA2, APOA5, and APOC3), as well as those involved in fatty acid transport and oxidation (e.g. FABP1, FABP3, ACS, ACO, CPT1, and CPT2), high-density lipoprotein (HDL) metabolism (e.g., PLTP), and ketone synthesis (e.g., HMGCS2), which take place in mitochondria, peroxisomes, and microsomes (22, 26, 27). Upon the activation of PPAR-α, the effect of substantial serum triglyceride clearance and HDL cholesterol increase is jointly yielded, as well as energy production. Moreover, PPAR-α also plays a key role in starvation response, which is underpinned by growing evidence on its regulation of carbohydrate and amino acid metabolism.

The catabolic action of PPAR-α on fatty acids causes an increased flux of fatty acids from peripheral tissues (e.g., skeletal muscle and adipose tissue), of which high content of triglyceride is significantly related to insulin resistance, to the liver. This effect might also consequently alleviate the FA-mediated inhibition of insulin-stimulated oxidative and non-oxidative glucose disposal in skeletal muscle, thus ameliorating insulin resistance. Additionally, the induction of hepatic lipogenesis of PPAR-α cooperates with insulin via regulating the sterol regulatory element-binding protein-1c (SREBP1c)-dependent pathway, a transcription factor modulating lipogenic enzyme expression and insulin action on both carbohydrates and lipids (28).

### Inhibition of Glycolysis

Cancer cells are characterized by their unique metabolic pathway, namely the Warburg effect, which in essence is glycolysis in oxygen-sufficient microenvironment. Under high energy demand and excessive oxidative stress, some cancer cells even adopt a hybrid metabolic method, employing both glycolysis and oxidative phosphorylation (OXPHOS). Upon activation, PPAR-α restricts mitochondrial OXPHOS, which non-tumor cells are not affected by; and increases the production of reactive oxygen species (ROS), causing the accumulation and exacerbation of the oxidative stress in cancer cell mitochondria. There is also a promotion of AMP-activated protein kinase (AMPK) signaling pathway witnessed alongside the activation of PPAR-α (29, 37), which increases the oxidation of fatty acids while potently inhibiting glycolysis, further cutting down ATP production. Synergistically, the mitochondria are impaired both structurally and functionally. Thus, the crippled energy production fails to meet the intensive energy demand of highly proliferative cancer cells, consequently impairing the viability, and even inducing cell cycle arrest, antagonizing the growth and metastasis of cancer (61).

### Promotion of Ketosis

Moreover, the prioritization of β-oxidation from PPAR-α as a starvation response has been illustrated. Upon agonist-binding, a decrease in urea concentration, an indicator of amino acid metabolism rate, is accompanied by an increase in ketone body concentration (30). Ketone bodies are of no energy-providing use for most cancer cells; however, for melanoma, the ketogenic gene expression is pro-tumorigenic, which is characterized by intracellular acetoacetate accumulation. Interestingly, acetoacetate is quite rapidly converted to beta-hydroxybutyrate in circulations and tissues within human body, and the latter suppresses inflammation, by the low-grade of which cancer microenvironment is always characterized, rather than immunosuppression. As the main regulator of physiological response to fasting and ketone body biosynthesis, PPAR-α has promising potential in the application in melanoma therapies (29).

### Regulation of Inflammation and eNOS

Tissues highly involved in global metabolism (e.g., adipose tissue, liver, skeletal muscle, and vascular walls) are prone to inflammation when there is a metabolic disturbance (62). Chronic low-grade inflammation and metabolic disorders in lipid and glucose homeostasis often co-exist, which makes PPAR, the pleiotropic metabolism regulator, a strong candidate in the modulation of innate immunity in various metabolic diseases. PPAR-α directly or indirectly exerts an anti-inflammatory effect: the activation by leukotriene B4 limits its own activity and attenuates inflammatory response via negative feedback; stimulated by PPAR-α, the increased gene expression of IκB, an NF-κB inhibitor, together with the protein-protein interaction of PPAR-α with p65 and c-Jun, downregulates the activator protein-1 (AP-1) and NF-κB pathway and thus interferes with proinflammatory activity (22, 33); and the interaction of PPAR-α with glucocorticoid receptor α or estrogen receptor also trans-represses other proinflammatory transcription factors (22).

In addition, upon activation, PPAR-α plays a significant role in the regulation of endothelial nitric oxide synthase (eNOS) in a non-metabolic way (34). The sustainability of eNOS translates to stable production of nitric oxide, a vasodilator, and anti-thrombotic agent protecting epithelium, of which the activity is severely compromised in patients with cardiovascular diseases and arthrosclerosis (31).

### Suppression of Angiogenesis

Since ample blood supply is critical to tumor growth, angiogenesis is determinant in the progression of tumor, involving degradation of the surrounding matrix, cell proliferation, migration, differentiation, and tube formation. In the NADPH oxidase (NOX) family, as NOX2, NOX4, and NOX1 all expresses in endothelial cells, NOX2, and NOX4 are involved in cell proliferation, while NOX1 mediates endothelial cell migration and sprouting but not proliferation (35). PPAR-α upregulates the gene-expression of endostatin and thrombospondin 1, which are angiogenic inhibitors; and downregulates vascular endothelial growth factor (VEGF); and cytochrome P450-2C (CYP-2C), the neovascularization induced by the former *in vivo* and *in vitro* is reported to be dependent on NOX2 (22, 35). Interestingly, PPAR-α has also been found as a downstream modulator in NOX1-mediated angiogenesis, whose activity is repressed by the presence of NOX1; and in NOX1-deficient cells, the upregulated-expression of PPAR-α blocks angiogenic signaling needed in endothelial cell migration, sprouting, and angiogenesis (35).

### Modulation of Immune System

Immunotherapy has been gaining significant momentum in cancer treatment, employing vaccines, antibodies, T cells, and cytokines to target the immune system to curb the growth of tumor cells. The valuable asset of metabolism-regulating of PPAR-α has established tight linkage to the generation, persistence, conversion, and apoptosis of T cell, of which the metabolic pathways play pivotal role in whose function and survival, greatly effecting the efficacy and outcome of the application. It is well-established that T-eff cells employ the classic metabolic mode—aerobic glycolysis—to sustain and recover effector function, which is the conversion from long-surviving memory cells to effectors (36); while T-memory cells majorly depend on fatty acid oxidation (FAO) and OXPHOS of mitochondria for energy.

According to studies, however, it is shown that in tumor microenvironment, with the metabolic constrains of hypoglycemia and hypoxia, due to the glucose depletion caused by tumor cells, which adopts glycolysis for energy production (63), T-effector cells perform better tumoricidal effect with increased mitochondrial metabolism, including OXPHOS, and FAO (64). It has been suggested that upon ligand-binding, PPAR-α, either working downstream in the activation of PPAR-δ by a PPAR-δ-specific ligand, GW501516 (65), or directly activated by co-activators (66), improves the efficacy of adoptive cell therapy by enhancing expression of carnitine palmitoyl transferase 1a, the rate-limiting enzyme of FAO, thereby enriching the uptake and oxidation of fatty acids. During which the expression of B-cell lymphoma-2 (Bcl2) is also upregulated, and the duo of the above two proteins can form a complex with the cytotoxic T lymphocytes (CTL) to exert an apoptosis-preventing effect (66). The activation of PPAR-α also improves anti-tumor immunity in PD-1 blockade cancer immunotherapy by reprogramming CD8+ T-cell metabolism from glycolysis to increased mitochondrial OXPHOS and FAO, supporting the extra energy demands of effector CTLs, thus lengthening the survival and potentiating activity (65, 66). Meanwhile, it also elevates cytokine expression (e.g., IFNγ) (64).

### Promotion of Tumorigenesis

However, the pro-tumorigenesis effect of PPAR-α has also been contended with some solid evidence. With regard to its powerful oxidative property, contrary to the aforementioned anti-tumorigenesis effect with excessive oxidative stress on cancer cell mitochondria, other scientists argued that the inhibition of PPAR-α has yielded anti-proliferative effect on human paraganglioma, pancreatic and colorectal cancer cells *in vitro* with decreased antioxidant capacity and carnitine palmitoyl transferase-1A pattern expression (49–52). As suggested before, there exist interactions between PPAR-α and hormone metabolism. Upon activation, PPAR-α increases the expression and activity of CYP1B1, a subtype of Cytochromes P450. Through the biotransformation of endogenous estrogens and environmental carcinogens, it is critical in the initiation and progression of various hormone-dependent tumors, including breast cancer (53). Under long-term administration, the activation of PPAR-α is found to be hepatocarcinogenic in rodents, a mechanism related to the downregulation of let-7c micro RNA expression, which stabilizes MYC mRNA, contributing to increased mitogenic signaling and the consequent hepatocyte proliferation. This is an effect via both PPAR-α-dependent and -independent pathway, which has been testified to be absent in humans (54, 55). Cancer stem cell (CSC) is a subset of cancer cell population possessing self-renewal ability, and its sphere-formation rate is positively correlated with the advancement of malignancy. The higher number of CSCs population, the greater potential tumor possesses to advance. It has been found that maintenance of CSC properties of human hepatocellular carcinoma cells is upregulated by PPAR-α pathway activation, through activation of the PPAR-α- stearoyl-CoA desaturase-1 axis (56).

## PPAR-α Modulators and Cancer

PPAR modulators including agonists and antagonists could represent a novel strategy for preventing and treating multiple types of cancer, regarding that dyslipidemias, obesity, glucose intolerance, and low-grade inflammation are strongly related to an increased risk of cancer, which PPAR modulators are able to directly or indirectly regulate. Thus, they are associated with cancer cell proliferation, differentiation, and apoptosis, supporting the potential of PPAR modulators as antitumor molecules. As far as PPAR-α agonists, they play an important role in the prevention of different cancers, including breast cancer, lung cancer, pancreatic cancer, and etc., by inhibiting the proliferation of cancer cells and affecting the Warburg effect. However, PPARs function as tumor suppressors or inducers is context-dependent, excessive expression of PPAR-α has been related to the progression of cell growth and survival in several cancer, suggesting that PPAR-α antagonists could be an effective therapeutic option for treating cancer (Table 2).

**Table 2:** Summary of the PPAR-α modulators in different types of cancer.

**Disease**	**Modulator**	**Agent**	**Main findings**	**References**
Head and neck paragangliomas	Antagonist	GW6471	Decreasing HNPGLs cells viability and proliferation by inhibiting PI3K/GSK3β/β-catenin pathway.	(67)
			Attenuating MMPs expression, enhancing AMPK phosphorylation, and suppressing NF-κB p65 and its DNA binding activity	(68)
Oral cancer	Agonist	Fenofibrate	Regulating the gene expression of mitochondrial energy metabolism	(69)
			Restraining the process of preneoplastic lesion to oral squamous cell carcinoma, downregulating mTOR activity, and adjusting Warburg effect to mitochondrial oxidative phosphorylation	(37)
Esophageal cancer	Agonist	Fenofibrate	Decreasing the expression of VEGF protein and sensitizing the cell to radiotherapy	(70)
Breast cancer	Agonist	Clofibrate	Regulating inflammatory, lipogenic pathways, and expression of genes involving FAO	(71)
		Fenofibrate	Reducing the phosphorylation levels of Akt/NF-κB and augmenting chemosensitivity when it combined with paclitaxel, TRAIL, ABT-737, and doxorubicin	(72)
		Fenofibrate	Arresting G1 cell cycle, restraining NF-κB activity and ERK signaling pathway	(73)
Lung cancer	Agonist		Preventing the progression of cancer anorexia cachexia syndrome	(74)
		Ave8134	Reducing the production of AA-derived EETs and promoting the levels of 11-HETE	(75)
Gastric cancer	Agonist	Fenofibrate	Reprogramming abnormal mitochondria via CPT-1 and FAO pathway and exhibiting trivial systematic toxicity	(32)
Pancreatic cancer	Agonist	Clofibrate	Promoting radiosensitivity of pancreatic cancer cells	(76)
	Antagonist	Sulfonimide derivative 4	Impairing viability in pancreatic cancer cells	(52)
		Fenofibrate	Inducing human HepG2 cell death by promoting the activity of ROS and intracellular glutathione depletion	(77)
Liver cancer	Agonist		Independently inhibiting human Huh7 cell proliferation without affecting by the PPAR-α antagonist (GW6471) or by PPAR-α-specific siRNA	(78)
		Clofibrate	Causing apoptosis or blocking proliferation in a time- and concentration- dependent way in human HepG2 cells	(79)
Prostate cancer	Agonist	Fenofibrate	Promoting cell autophagy in the beginning but inhibiting complete autophagy eventually	(80)
			Interfering energy metabolism and impairing microevolution and expansion induced by drug-resistant cells	(81)
		LY171883	Diminishing AP-1-mediated transcriptional activation of genes involving inflammatory response like Cox-2 and VEGF	(82)
		WY14643	Diminishing AP-1-mediated transcriptional activation of genes involving inflammatory response like Cox-2 and VEGF	(82)
Colorectal cancer	Agonist		Increasing chemosensitivity and inhibiting mTOR pathway	(38)
		Clofibrate	Inducing antiapoptotic Bcl2 protein degradation and promoting autophagy	(83)
		Fenofibrate	Decreasing expression of DNMT1 and PRMT6	(39)
	Agonist	Fenofibrate	Alleviating glycolysis and lactate production, and impairing mitochondrial respiration in glioblastoma cells	(84, 85)
			Distinct reactions of glioblastoma cells could be obtained under different doses of fenofibrate	(86)
Glioblastomas		AA452	Decreasing cholesteryl esters and lipid droplets, reprograms lipid metabolism, regulation of MVA pathway	(87)
	Antagonist	MK886	Inhibiting 5-LO expression, and blocking ERKs phosphorylation and activation of Bcl-2/Bax signaling	(88)
			Overcoming TRAIL resistance to enhances apoptosis in glioma cells	(89, 90)
Chronic lymphocytic leukemia	Antagonist	MK886	Causing proliferating CLL cells to access immunogenic death pathway and directly inducing apoptosis of circulating CLL cells	(91)
		NXT629	Inducing CLL cells death even in the presence of a protective microenvironment	(92)
Acute myeloid leukemia	Agonist	Bezafibrate	The hematological scores of 4 participants improved, and no disease progression in remaining 7 subjects	(93)
Endemic Burkitt lymphoma	Agonist	Bezafibrate	Disease progression was 29%, 0%, and 0% in low-, intermediate-, and high-dose groups respectively	(94)
Melanoma	Agonist	Fenofibrate	Inhibiting melanogenic apparatus to reduce total melanin content in melanoma cells	(95)
			Inhibiting the expression of TLR-4, MyD-88 and NF-κB gene	(96)
Angiosarcomas	Agonist	Fenofibrate	Arresting cells in G2/M cell cycle phase, hyperpolarizing mitochondria, and downregulating the expression of VEGF-dependent ‘oncoproteins’ including Akt, survivin, ERK and Bcl-2	(97)

*HNPGL, head and neck paragangliomas; PI3k, phosphatidylinositol 3-kinase; GSK3, glycogen synthase kinase3; MMPs, matrix metalloproteinases; AMPK, adenosine monophosphate -activated protein kinase; NF-κB, nuclear factor κB; mTOR, rapamycin; VEGF, vascular endothelial growth factor; FAO, fatty acid oxidation; Akt, protein kinase B; TRAIL, tumor necrosis factor-related apoptosis-inducing ligand; ERK, extracellular regulated protein kinases; EETs, epoxyeicosatetrienoic acids; HETE, hydroxyeicosatetraenoic acid; CPT, carnitine palmitoyltransferase; ROS, reactive oxygen species; AP-1, activator protein-1; Cox-2, cyclooxygenase-2; Bcl-2, B-cell lymphoma-2; DNMT1, DNA (cytosine-5-)-methyltransferase-1; PRMT6, protein arginine N-methyltransferase; 5-LO, 5-lipoxygenase; CLL, chronic lymphocytic leukemia; TLR-4, Toll-like receptor-4; MyD-88, myeloid differentiation factor 88*.

### Head and Neck Paragangliomas

Head and neck paragangliomas (HNPGLs) are rare types of cancer that lead to significant morbidity regarding their ability to infiltrate the skull base (98). At present, surgery is the only option for patients, whereas, it is difficult to thoroughly remove the tumors (99). Therefore, other effective therapies are highly needed. Considering that PPAR-α is a candidate treatment for several types of cancer, a study has analyzed the expression of PPAR-α nuclear receptor in HNPGLs and assessed the functions of two PPAR-α modulators: PPAR-α agonist (WY14643) and PPAR-α antagonist (GW6471) in a unique model of HNPGLs cells, where the protein expression level of PPAR-α is remarkably high. The study has demonstrated that PPAR-α agonist (WY14643) fails to affect HNPGLs cells viability, while PPAR-α antagonist (GW6471) is associated with the decrease of HNPGLs cell viability and proliferation. Moreover, the underlying mechanisms involve the inhibition of the PI3K/GSK3β/β-catenin pathway, resulting in interfered cell cycles and induced apoptosis, resulting in the inhibition of clonogenicity and migration of HNPGL cells (67). Thus, PPAR-α antagonist (GW6471) could be considered as a potential therapy for HNPGLs.

### Oral Cancer

Oral cancer is the one of the fatal malignancies with frequent lymph node metastasis and local invasion, thus, the prognosis of patients is poor even after targeted and chemotherapeutic drugs (100). Fenofibrate, a PPAR-α agonist, has been widely used to treat hyperlipidemia with its effects of anti-inflammatory and anti-atherosclerotic in humans. Meanwhile, the anticancer potential of fenofibrate has been report in several studies, involving induction of cancer cell apoptosis, decrease of cell proliferation, suppression of tumor angiogenesis, and inhibition of oxidative stress (101, 102). A study has investigated the anticancer activities of fenofibrate on the invasion and migration of human oral cancer Cal27 cells, which is associated with the attenuation of MMPs expression, enhancement of AMPK phosphorylation, and suppression of NF-κB p65 and its DNA binding activity, while such effect was observed to present in a AMPK-dependent manner, rather than PPARα signaling (68). The same research team has also found that in oral cancer mouse model, the expression level of PPAR-α protein was negatively correlated to cancer advancement; and the activation of PPAR-α by fenofibrate induced decreased migration ability in oral cancer cells *in vitro*, assumably via reprogramming ATP pathway, interfering with the characteristic Warburg effect of cancer cells (69). Moreover, in both animal and cell culture models, fenofibrate shows anti-oral cancer effects in restraining the process of preneoplastic lesion to oral squamous cell carcinoma, downregulating mTOR activity via TSC1/2-dependent signaling by triggering AMPK and suppressing Akt, and adjusting Warburg effect to mitochondrial oxidative phosphorylation to control energy production approach so as to repress proliferation of oral cancer cells and induction of metabolic reprogramming (37).

### Esophageal Cancer

Esophageal cancer ranks as the sixth leading cause of cancer-related death worldwide. According to studies, there is a negative correlation between VEGF expression and cancer cell radiosensitivity, and the inhibition of VEGF expression promotes radiosensitivity of esophageal cancer cells, while the administration of fenofibrate can effectively diminish hypoxia-induced VEGF secretion in MCF-7 cells, a mechanism whose association with PPAR-α expression level remains unidentified. Either the combination of fenofibrate and radiation or fenofibrate alone is able to significantly decrease the expression of VEGF protein (70). Furthermore, a synergistic effect was observed in the combined administration of fenofibrate and radiation, which induced higher ratio of cells in G2/M phase and suppressed the growth of esophageal cancer cells (70).

### Breast Cancer

Breast cancer is the second cause of cancer-related death in women (103), chemotherapy functions in preventing the progression of the primary breast cancer in neoadjuvant setting, but it may lead to several side effects and apoptosis resistance in breast cancer patients (104). Among fat and triglyceride lowering drugs, clofibrate, fenofibrate and WY14643 (a 2-aryl-thioacetic acid analog of clofibrate), PPAR-α agonists, present high chemosensitivity toward breast cancer cells. Clofibrate is the first lipid-lowering fibric acid derivative. According to Chandran et al. (71) the administration of clofibrate to breast cancer cells, the expression level of PPAR-α in which was abnormally high, showed significant cytotoxicity. The anti-carcinogenic effect was achieved possibly via the induction of PPAR-α DNA binding activity, causing cell cycle arrest, and the reduction of signaling, lipogenic, and inflammatory pathways, causing the suppressed proliferation of breast cancer cells (105). More specifically, researchers have the supposition that clofibrate suppresses the growth of breast cancer cells by repressing NF-κB and extracellular regulated protein kinases1/2 (ERK1/2) activation, inhibiting cyclinD1, cyclinA, cyclinE, and inducing pro-apoptotic P21 levels. Also, clofibrate significantly reduces Cox-2/PGE2(phenyl glycidyl ether-2) and 5-lipoxygenase/LTB4 (leukotriene B4) inflammatory pathways. Besides, clofibrate effectively mediates the expression of lipogenic and FAO pathways genes including SREBP-1c, SREBP-2, HMG-CoA synthase 2, Acyl-CoA oxidase, and CPT-1a (carnitine palmitoyltransferase 1a) (71). These findings provide new insight into our understating of clofibrate as a therapeutic anticancer agent.

In the process of cell apoptosis, the activation of Akt/NF-κB pathway plays a pivotal role in inhibiting the major apoptotic regulators, which hinders the activity of pro-apoptosis, are Bax, Bok, and Bim, leading to resistance of drug-induced cell death (106, 107). Fenofibrate is capable to potentiating chemosensitivity in breast cancer treatment by regulating Akt/NF-κB pathway, which is responsible for apoptosis resistance in some breast cancer patients. It is reported that fenofibrate promotes chemosensitivity by remarkably reducing the phosphorylation levels of Akt/NF-κB, as well as the downregulation of Mcl-1 and Bcl-xl and the upregulation of Bok and Bax at transcription level. Meanwhile, the study has indicated that the activation of caspase-9 and caspase-3 and the permeabilization of mitochondrial outer membrane affect fenofibrate-elevated chemosensitivity. Moreover, the synergistic effects of fenofibrate with paclitaxel, tumor necrosis factor-related apoptosis-inducing ligand (TRAIL), ABT-737, and doxorubicin significantly augment chemosensitivity to enhance the apoptosis of breast cancer cells (72). Basing on the emerging evidence, fenofibrate is a promising candidate in breast cancer treatments.

### Lung Cancer

Lung cancer remains the most common cancer in the world, with a high mortality and a poor 5-year survival rate, even after radiation and immunotherapy (108). However, there is promising evidence that the PPAR-α agonist, fenofibrate, and the glucocorticoid, budesonide have been found to be beneficial to lung cancer (109, 110). A study used two types of lung adenocarcinoma cells: A549 (wild type TP53) and SK-MES-1 (TP53 deficient) to evaluate the effects of budesonide and fenofibrate, alone and in combination, which showed differential effects on the growth of lung cancer cells, in a PPAR-α-independent fashion. Budesonide inhibits cell proliferation in wild type TP53 A549 cells, whereas, this anti-proliferation effect is abrogated in TP53 deficient SK-MES-1 lung cancer cells. However, fenofibrate shows anti-proliferation effect in both A549 cells and SK-MES-1 cells. Furthermore, in A549 lung cancer cells, there is an additive effect against cell growth when using the combination of budesonide and fenofibrate, indicating a better therapeutic effect could be obtained with the combination of two compounds. The inhibition of cell growth induced by budesonide and fenofibrate is associated with G1 cell cycle arrest and the restraint of NF-κB activity and ERK signaling pathway (73). Besides, fenofibrate is able to prevent the progression of cancer anorexia cachexia syndrome (CACS) which is characterized by body weight loss, reduced food intake, and the catabolism of stored nutrients in muscle and adipose tissue. In lungs of CACS mice, the expression level of PPAR-α was reduced both in the nucleus and cytoplasm, compared to non-CACS counterparts. After the treatment of fenofibrate, the upregulation of the expression levels of several PPAR-α target genes, including Hmgcs2, Acadm, Cyp4a14, Acox1, and Ehhadh, has been observed. This effect may be related to improved FAO by facilitating peroxisome proliferator activity in the lungs (74).

In addition, a novel PPAR-α agonist, Ave8134, is well-tolerated in humans and shows advantages in cancer treatment. Ave813 suppresses the expression of CYP2c44, a functional homolog of human CYP2c9 that composes the major CYP epoxygenases for epoxyeicosatrienoic acids (EETs) biosynthesis in endothelial cells. Previous studies have confirmed that inhibiting the expression of CYP2c44 gene decreases endothelial proliferation and tumor growth. With the reduction of CYP2c44 expression, EET synthesis weakens, leading to reduced angiogenesis, and declined development of tumor (111, 112). However, there is conflicting evidence that Ave813 prominently promotes the levels of 11-hydroxyeicosatetraenoic acid (11-HETE), a bioactive lipid mediator converted by arachidonic acid (AA), which stimulates angiogenesis and tumor progression. Nevertheless, a Cox inhibitor indomethacin can effectively block the production of 11-HETE (75), thus the combination of indomethacin and AVE8134 may have promise in treatments for lung cancer.

### Gastric Cancer

Gastric cancer has a low rate of early diagnosis, and advanced gastric carcinoma is mainly treated by systematic chemotherapy, leading to serious adverse effects (113). PPAR-α is overexpressed and inversely related to prognosis of gastric cancer. Fenofibrate was shown to be capable of reprogramming abnormal mitochondria via CPT-1 and FAO pathway, as well as stimulating the AMPK signaling and suppressing the hexokinase-2 (HK-2) signaling. As a result, it is able to regulate the metabolism of glucose and lipid, prevent the growth of gastric cancer cells, and trigger the apoptosis of gastric cancer cells. Besides, fenofibrate also exhibits trivial toxicity in gastric tumor mouse model (32). It is expected that more investigations should be carried out on the effect of fenofibrate or other PPAR-α modulators in gastric cancer given the scarce existing evidence.

### Pancreatic Cancer

Pancreatic cancer remains as one of the most intractable and devastating types of cancer in the world, and few clinical development has been achieved for pancreatic cancer in the past decade, with surgery and chemotherapy being major curative treatments (114). A study used pancreatic cancer tissue samples from human to assess the level of PPAR-α in different pancreatic cancer tissues. The expression of PPAR-α is considerably lower in normal adjacent tissues than in pancreatic cancer tissues, which is strongly related to the prognosis of pancreatic cancer patients. Also, the activation of PPAR-α by its agonist, clofibrate, promotes radiosensitivity of pancreatic cancer cells via downregulating PTPRZ1 and Wnt8a transcription (two crucial components of Wnt/β-catenin pathway). This effect is abrogated by PPAR-α antagonist GW6471 and PPAR-α silencing, indicating a PPAR-dependent manner (76). Additionally, another study has examined the effects of a novel sulfonimide derivative 4 with PPAR-α antagonistic feature and a weaker PPAR-γ antagonism, and the novel compound potently impairs the viability of pancreatic cancer cell lines, indicating an inhibition of PPAR-α and PPAR-γ could be a therapeutic option for pancreatic cancer (52).

### Liver Cancer

Hepatocellular carcinoma is one of the leading causes of cancer death in Asian countries. Unlike advanced breast cancer, PPAR-α expression is reduced in hepatocellular carcinoma, and PPAR-α agonists show anticancer property in liver tumor (115). In hepatocarcinoma HepG2 cells, fenofibrate and clofibrate induce cell apoptosis in a dose-related manner. Due to the different level of PPAR-α in human and rodent liver that human is significantly less than rodent, chronic application of fenofibrate causes liver cancer in rodent, however, high concentration of fenofibrate induces human HepG2 cells death by promoting the activity of ROS and intracellular glutathione depletion (77). Furthermore, clofibrate causes apoptosis or blocks proliferation in a time- and concentration- dependent way in human HepG2 cells by increasing protein phosphatase-2A expression and the pro-apoptotic BAD (79).

In hepatocarcinoma Huh7 cells, fenofibrate is able to independently inhibit human Huh7 cells proliferation without affecting by the PPAR-α antagonist (GW6471) or by PPAR-α-specific siRNA. Fenofibrate triggers the expression of C-terminal modulator protein, leading to decreased Akt phosphorylation which stimulates the nuclear accumulation of cyclin-dependent kinase inhibitor p27. More accumulation of p27 and reduction of cyclin A and E2F transcription factor 1 cause G1 arrest, eventually contributing to human Huh7 cells death (78). Thus, the antiproliferative property of fenofibrate and clofibrate makes them potential for anti-hepatoma therapy.

### Prostate Cancer

The most common cancer of genital system among males is prostate cancer (116), and based on the existing evidence, fenofibrate may plays a crucial role in the management of prostate cancer. A study has investigated the effects of fenofibrate prostate cancer cells, where higher rate of apoptosis was observed after the administration of fenofibrate compared to the control group. Also, the study has found that fenofibrate promotes autophagy in the beginning but inhibits complete autophagy eventually by adjusting AMPK-mTOR pathway, which leads to increased ER stress via PERK and IRE1 pathways, and the cumulation of ER stress accelerates cell death. Meanwhile, it has been observed that fenofibrate significantly inhibits the growth of prostate tumor *in vivo* mice model (80).

In order to examine the effect of fenofibrate on the drug-resistance of prostate cancer cells, another study applied the combined treatment of docetaxel/mitoxantrone and fenofibrate to naïve and drug-resistant cells. As expected, fenofibrate increases the chemosensitivity of prostate cancer cells by interfering energy metabolism and impairing microevolution and expansion induced by drug-resistant cells (81). Noteworthily, these effects can be obtained in the presence of <25 μM fenofibrate, i.e., within the range of its tolerable serum concentrations (up to 100 μM) (117).

### Colorectal Cancer

The morbidity of colorectal cancer is showing the tendency of increasing, with one of the important risk factors being dietary fat. Therefore, the regulation of systemic lipid homeostasis plays a key role in controlling the development of colorectal cancer, which may be achieved by the application of PPAR-α modulators (118). In colorectal carcinoma SW620 cells which express low levels of PPAR-α mRNA, two PPAR-α agonists, LY171883, and WY14643 attenuate early stages of colon tumorigenesis by diminishing AP-1-mediated transcriptional activation of genes involving inflammatory response like Cox-2 and VEGF via PPRE-driven transcription in a PPAR-α-dependent fashion (82). Moreover, WY14643 is able to increase chemosensitivity via affecting the transcriptional activity of glucose transporter-1, and inhibit mTOR pathway, leading to apoptosis of cancer cells (38). With regards to colorectal carcinoma SW480 cells, another PPAR-α agonist, clofibrate, significantly suppresses tumor proliferation and sensitizes SW480 cells to chemotherapy drugs in a PPAR-α-dependent manner that induces antiapoptotic Bcl2 protein degradation and promotes autophagy (83). Furthermore, PPAR-α transgenic mice with increased expression of DNMT1 and PRMT6 have higher susceptibility to the development of colorectal cancer, which can be reduced by the activation of PPAR-α with the application of fenofibrate (39). Based on the evidence available, PPAR-α agonists are posed as potential drugs in the treatment for colorectal cancer.

### Glioblastomas

Glioblastomas are the most malignant and incurable brain tumors characterized by rapid proliferation, resistance to radio- and chemotherapy, and persistent invasion of the central nervous system. The rapid growing cancer cells require both large amounts of ATP generated by mitochondrial respiration and glucose carbons produced by glycolysis (119, 120). Thus, disturbing those pathways could be a therapeutic strategy for glioblastomas, and several studies have demonstrated fenofibrate's capability of selectively promoting metabolic catastrophe in glioblastoma cells through interfering with mitochondrial function and glucose uptake. A study has detected the effects of fenofibrate in two settings: in a PPAR-α-independent manner, the unprocessed fenofibrate represses mitochondrial respiration; and in a PPAR-α-dependent manner, fenofibric acid converted by blood and tissue esterases stimulate glioblastoma cells to switch from glycolysis to FAO. These effects attenuate intracellular ATP and promote the AMP-activated protein kinase, which leads to extensive glioblastoma cells death. Interestingly, the study has also found that autophagy activators decrease the cytotoxicity of fenofibrate, while autophagy inhibitors increase the fenofibrate-induced glioblastoma cytotoxicity including phosphorylation of AMPK and suppression of mTOR- dependent phosphorylation of p70S6K (84). Similar findings were acknowledged in another study where fenofibrate not only alleviates glycolysis and lactate production but also impairs mitochondrial respiration in glioblastoma cells by inhibiting the transcriptional activity of NF-κB/ RelA and disrupting its association with HIF1α (85). The role of NF-κB/ RelA has been intensively studied, which is related to cancer cell growth, angiogenesis, and metastasis (121), as well as triggers aerobic glycolysis through transcriptional activation of pyruvate kinase isozyme type M2 (PKM2) (122). Moreover, the high ratio of PKM1/PKM2 enhances glycolysis and inhibits oxidative phosphorylation, whereas, fenofibrate is able to reduce the PKM2/PKM1 ratio and result in mitochondrial dysfunction (85).

In addition, distinctively differential reactions from glioblastoma cells could be observed with different doses of fenofibrate applied. Compared to a dose of 25 μM fenofibrate, 50 μM fenofibrate induces massive apoptosis of glioblastoma cells, while 25 μM fenofibrate inhibits monolayer and clonogenic growth of the tumor, and merely leads to trivial cell death, which is associated with the accumulation and phosphorylation of forkhead box O-3A (FoxO-3A), as well as the increase of FoxO-dependent apoptotic protein, Bim (86). Although the anticancer property of fenofibrate has been widely acknowledged, a dilemma that fenofibrate hardly crosses blood brain barrier makes it less effective in promoting glioblastoma cells death. According to one study, a novel synthesized compound PP1 with similar chemical structure of fenofibrate, but more stable, water soluble, and tissue penetrable, causes extensive glioblastoma cells death without showing major signs of distress (123).

AA452 is another PPAR-α antagonist tested for the treatment of glioblastoma, which showed encouraging affects in regulating lipid metabolism and radiosensitivity of glioblastoma cells. It decreases cholesteryl esters and lipid droplets and reprograms lipid metabolism, in parallel with the regulation of mevalonate (MVA) pathway, consequently limiting cancer cells proliferation and migration, as well as decreasing the invasiveness of glioblastoma cells. This inhibition of cholesteryl esters, from AA452, is truly promising since cholesteryl esters play an important role in malignancy. Moreover, AA452 also sensitizes glioblastoma cells to radiotherapy, leading to more cell death, whose mechanism is associated with the downregulation of cyclinD1 protein and c-myc gene (87).

5-LO is the key enzyme that catalyzes the first two steps in the synthesis of all leukotrienes by AA dioxygenation, and leukotrienes have significantly higher expression levels in brain tumor tissues than in normal brain tissues. Also, 5-LO plays an essential role in the signaling via Ras-ERKs pathway which is associated with the growth and progression of brain tumor. Therefore, the inhibition of 5-LO is beneficial in brain tumor treatment (124). A study used human glioma cells U-87MG and A172 with strong expression of 5-LO, and 5-LO non-expressing cells U373 to detect the anticancer effects of a PPAR-α antagonist—MK886. The study has found that MK886 is a specific 5-LO inhibitor with a high dependence of 5-LO expression level, and that MK886 induced cytotoxicity in cells U-87MG and A172 but not in cells U373, with its antiproliferative property possibly linked to the blocking of ERKs phosphorylation and activation of Bcl-2/Bax signaling (88).

The TRAIL (TNF-related apoptosis-inducing ligand) selectively induces apoptosis in transformed cells without killing most normal cells, rendering it a promising candidate in brain tumor treatment. However, the resistance of TRAIL impedes its application in cancer therapy. Thrillingly, a study showed that MK886 may possess the ability to overcome TRAIL resistance, enhancing its apoptotic effect on glioma cells and suggesting a combination strategy, which may exert more potent effect than each agent alone (89). Moreover, the research team has also revealed the mechanisms of sensitization of TRAIL-induced apoptosis by MK886: it was found that MK886 reduced the expression of an antiapoptotic protein, namely survivin, which is related to tumor cell resistance to TRAIL, and MK886 activates p38 mitogen-activated protein kinase (MAPK) pathway, leading to the overexpression of the death receptor 5 (DR5), eventually causing intensive apoptosis in glioma cells (90).

### Blood Cancer (Leukemia, Lymphoma, and Myeloma)

Chronic lymphocytic leukemia (CLL) is the most common adult leukemia in the western countries awaiting advanced treatment (125). Unlike acute leukemias, CLL cells mainly generate energy through FAO, rather than Warburg effect and glycolysis. According to Spaner et al. (91), measured by real-time PCR, the mRNA expression levels of PPAR-α in circulating CLL cells from two patients were present and almost 3-fold higher than that of peripheral blood mononuclear cells from a normal donor. As a major transcriptional regulator of FAO, the PPAR-α antagonists may be promising for CLL treatment. It can be observed in the study that, MK886 not only causes proliferating CLL cells to access immunogenic death pathway, but also directly induces apoptosis of circulating CLL cells, which is related to the reduction of interleukin-10 (IL-10) and phospho-STAT3 (91). Compared with MK886, NXT629 is more selective in killing CLL cells, and it is able to induce CLL cells death even in the presence of a protective microenvironment (92). Although NXT629 shows curative effects of CLL *in vitro* and *in vivo*, its pharmacokinetic properties for further clinical development have not been identified to date (126).

Current cytotoxic chemotherapy deteriorates the situation of reduced hemopoiesis in patients with acute myeloid leukemia (AML). Nevertheless, a small scale of clinical study has examined the safety and efficacy of the combinational therapy of bezafibrate (a PPAR-α agonist) and medroxyprogesterone acetate in 20 AML patients, and it was found that the low-dose combinational therapy showed no hematological toxicity and could be applied persistently in treating AML (93). However, the latter study has demonstrated that no extra benefit has been found with the same regime at a higher dosage in improving hemopoiesis or treating AML (127).

Children living in malaria-prone place are susceptible to endemic Burkitt lymphoma, and limited treatments restrict the cure rates (128). A clinical trial recruited 95 children with endemic Burkitt lymphoma to investigate the anti-endemic Burkitt lymphoma activity of three differential doses (low, intermediate, and high) of the combination of bezafibrate and medroxyprogesterone acetate. It was observed that the CCR (complete clinical response) of the high BaP (Bezafibrate and Medroxyprogesterone acetate) dose cohort was significantly higher at 68%, while that of the intermediate and low BaP cohorts showed relatively unremarkable difference, at 18 and 24%, respectively (129). Additionally, another study has verified that clofibrate reduces cell proliferation in multiple lymphoma and myeloma cells, which may be associated with Wnt pathway (94).

### Melanoma

Developing from melanocytes present in the epidermis, dermis, hair follicle of the skin, and other parts of the body, melanoma has an increasing morbidity worldwide in the past decades (130). In a study investigating the association of PPAR-α expression level and pigmentation of melanoma cells, which showed a inverse correlation, fenofibrate was observed to act as a depigmentation agent and suppresses the melanogenic apparatus in a PPAR-α-independent manner, which reduces total melanin content in B16 F10-derived cell lines (95). Additionally, fenofibrate can also exert anti-melanoma effects as a result of its significant anti-inflammatory effect, through inhibiting the expression of TLR-4, myeloid differentiation factor 88 (MyD-88), and NF-κB gene and the generation of TNF-α. However, the effect showed no dose-dependent pattern (96).

### Angiosarcomas

Angiosarcomas are lethal and aggressive soft tissue malignancies originating from endothelia. According to Majeed et al. (97) fenofibrate was observed to exhibit prominent anti-proliferative property independent of PPAR-α in VEGF-dependent angiosarcomas cells. It induced cell arrest in G2/M phase, hyperpolarizes mitochondria, and downregulates the expression of VEGF-dependent “oncoproteins” including Akt, survivin, ERK, and Bcl-2, without reducing viability or inducing apoptosis of angiosarcomas cells. Noteworthily, the effects are observed to be not abrogated by PPAR-α and NF-κB inhibitors, and their combination with fenofibrate was cytotoxic. Whereas, other PPAR-α agonists including bezafibrate, WY14643, and fenofibric acid fail to replicate the effects of fenofibrate (97).

## Discussion and Conclusion

Cancer remains one of the leading causes of morbidity and mortality globally, but PPAR-α modulators shows great capability to manage the process of cancer. Indeed, by analyzing research evidence from a number of studies, we have discussed the functions of PPAR-α to clarify the underlying mechanisms of PPAR-α modulators in treating different malignancies. PPAR-α regulates energy metabolism of lipid, glucose and protein, affects inflammation and eNOS, modulates immune response, and adjusts proliferation, differentiation and apoptosis of cancer cell. Simultaneously, possible PPAR agonists and antagonists that may hold potential treatment for cancer patients have been comprehended and provided in this review for comparison across the clinical strategies. However, PPAR-α presents pro-tumorigenesis property as well, indicating that PPAR-α may promote the progression of tumor, which has been demonstrated in several studies that PPAR-α agonists induced hepatocarcinoma or peroxisome proliferation in rodents (131). Also, studies have reported that exposures of weeks to several years of PPAR-α agonists treatment may induce hepatocellular chronic active and cholestatic hepatitis, as well as increase the level of serum transaminase, which may be dose related (132). Besides, fibrates have a risk of muscle astrophy, and the combination of fibrates and stains may also increase the risk of rhabdomyolysis (133). Other adverse effects include renal damage that fibrates may impair the generation of vasodilatory prostaglandins, leading to a reversible decrease in glomerular filtration rate and a elevation of serum creatinine (134, 135), and gallbladder disease such as gall stone, but the incidence varies remarkably between compounds (132). Therefore, routine monitoring of liver and renal function should be administrated in patients with PPAR-α modulators therapy. By establishing this thorough review on PPAR-α modulators, we hope to provide valuable insight to how we can better tackle cancer in the future.

## Author Contributions

ZL and PW: conceptualization and supervision. YT and PW: resources. KY, TC, and MW: writing—original draft preparation. TC, MW, YT, and ZL: writing—review and editing. PW: project administration. PW and YT: funding acquisition. All authors have read and agreed to the published version of the manuscript.

## Conflict of Interest

The authors declare that the research was conducted in the absence of any commercial or financial relationships that could be construed as a potential conflict of interest.

## Abbreviations

5-LO: 5-lipoxygenase
AA: arachidonic acid
ACO: acyl-CoA oxidase
ACS: acyl-CoA synthetase
Akt: protein kinase B
AMP: adenosine monophosphate
AMPK: AMP-activated protein kinase
AML: acute myeloid leukemia
AP-1: activator protein-1
APOA1: apolipoprotein A1
APOA2: apolipoprotein A2
APOA5: apolipoprotein A5
APOC3: apolipoprotein C3
Bcl2: B-cell lymphoma-2
CACS: cancer anorexia cachexia syndromes
CKD4: cyclin-dependent kinases4
CLL: chronic lymphocytic leukemia
Cox: cyclooxygenase
CPT: carnitine palmitoyltransferase
CSC: cancer stem cell
CTL: cytotoxic T lymphocyte
CYP: cytochrome P450
DNMT1: DNA (cytosine-5-)-methyltransferase-1
DR5: death receptor5
EETs: epoxyeicosatetrienoic acids
eNOS: endothelial nitric oxide synthase
ER stress: endoplasmic reticulum stress
ERK: extracellular regulated protein kinases
FABP1: fatty ccid-binding protein 1
FABP3: fatty ccid-binding protein 3
FA: fatty acid
FAO: fatty acid oxidation
FLAP: 5-lipoxygenase activating protein
FoxO: forkhead box O
FASN: fatty acid synthase
GSK3: glycogen synthase kinase3
HEI-OC1: house ear institute organ of corti1
HETE: hydroxyeicosatetraenoic acid
HK-2: hexokinase-2
HIF1α: hypoxia inducible factor-1α
HDL: high-density lipoprotein
HMGCS2: hydroxymethylglutaryl CoA synthase 2
HNPGLs: head and neck paragangliomas
IFNγ: interferon gamma
IκB: inhibitor of NF-κB
JNK: c-Jun N-terminal kinase
LTB4: leukotriene B4
MAPK: mitogen-activated protein kinase
MMPs: matrix metalloproteinases
mTOR: mammalian target of rapamycin
MSC: mesenchymal stem cells
MVA: mevalonate
MYC: avian myelocytomatosis virus oncogene homolog
MyD-88: myeloid differentiation factor 88
NADPH: nicotinamide adenine dinucleotide phosphate
NF-κB: nuclear factor κB
NLRP3: nucleotide-binding domain, leucine-rich-containing family, pyrin domain-containing-3
NOX: NADPH oxidase
OXPHOS: oxidative phosphorylation
PI3k: phosphatidylinositol 3-kinase
PKM1: Pyruvate kinase isozyme type M1
PKM2: Pyruvate kinase isozyme type M2
PRMT6: protein arginine N-methyltransferase
PD-1: programmed cell death protein 1
PGE: phenyl glycidyl ether
PLTP: phospholipid transfer protein
PPAR: peroxisome proliferator-activated receptors
ROS: reactive oxygen species
RXR: retinoid X receptor
SREBP: sterol regulatory element-binding transcription factor
STAT3: signal transducer and activator of transcription 3
TLR-4: Toll-like receptor-4
TRAIL: tumor necrosis factor-related apoptosis-inducing ligand
VEGF: vascular endothelial growth factor.
